# Antibiotic Potentiation as a Promising Strategy to Combat Macrolide Resistance in Bacterial Pathogens

**DOI:** 10.3390/antibiotics12121715

**Published:** 2023-12-11

**Authors:** Deepjyoti Paul, Meenal Chawla, Taruna Ahrodia, Lekshmi Narendrakumar, Bhabatosh Das

**Affiliations:** Functional Genomics Laboratory, Translational Health Science and Technology Institute (THSTI), NCR Biotech Science Cluster, Faridabad 121001, India

**Keywords:** antibiotic, antimicrobial resistance, azithromycin, macrolide resistance, multidrug resistance, potentiation, adjuvants, macrolide potentiator

## Abstract

Antibiotics, which hit the market with astounding impact, were once called miracle drugs, as these were considered the ultimate cure for infectious diseases in the mid-20th century. However, today, nearly all bacteria that afflict humankind have become resistant to these wonder drugs once developed to stop them, imperiling the foundation of modern medicine. During the COVID-19 pandemic, there was a surge in macrolide use to treat secondary infections and this persistent use of macrolide antibiotics has provoked the emergence of macrolide resistance. In view of the current dearth of new antibiotics in the pipeline, it is essential to find an alternative way to combat drug resistance. Antibiotic potentiators or adjuvants are non-antibacterial active molecules that, when combined with antibiotics, increase their activity. Thus, potentiating the existing antibiotics is one of the promising approaches to tackle and minimize the impact of antimicrobial resistance (AMR). Several natural and synthetic compounds have demonstrated effectiveness in potentiating macrolide antibiotics against multidrug-resistant (MDR) pathogens. The present review summarizes the different resistance mechanisms adapted by bacteria to resist macrolides and further emphasizes the major macrolide potentiators identified which could serve to revive the antibiotic and can be used for the reversal of macrolide resistance.

## 1. Introduction

Macrolides are one of the most clinically significant and widely prescribed drugs worldwide after β-lactams, used to treat both Gram-positive and Gram-negative bacterial infections [1]. During the COVID-19 pandemic, the use of macrolide antibiotics was greatly increased due to their efficacy in treating community-acquired respiratory tract infections, anti-inflammatory and immunomodulatory properties, though there were not sufficient studies evidencing that treatment with macrolides, alone or in combination, was effective in treating COVID-19 patients [2,3,4]. Overall, this extensive use acted as a strong selective pressure contributing to the development and expansion of macrolide-resistant determinants amongst both Gram-positive and Gram-negative bacteria [5]. Recently, the WHO classified antibiotic-resistant priority pathogens as critical, high, and medium priority based on the significance and severity of the infection caused by these organisms and the urgency of the need for new antibiotics against these pathogens. Unsurprisingly, this list included macrolide-resistant organisms as high-priority pathogens [6]. In times of increasing antibiotic resistance, it is of critical importance to study and understand the bacterial resistance mechanisms, which could help in the development of new drugs or find other alternative approaches that can help to reduce the emergence of resistant pathogens. Antibiotic potentiators represent a promising approach for restoring the efficacy of antibiotics against multidrug-resistant (MDR) pathogens. Antibiotic potentiators are compounds that do not themselves possess antibacterial properties, but when used in combination with antibiotics, enhance the antibacterial efficacy of the antibiotics. The resurgence of interest in discovering such molecules is primarily driven by the urgent need to revive the existing obsolete drugs and increase therapeutic options against MDR pathogens. [7]. Several reviews have emphasized antibiotic resistance and the significance of potentiators, but there is a notable absence of focus on macrolide antibiotics, despite their broad clinical use [8,9]. In this review, the authors summarize different mechanisms Gram-negative bacteria adopt for macrolide resistance, as well as the most recent discoveries and developments in the field of macrolide potentiators.

## 2. Resistance Mechanisms to Macrolide: Special Emphasis on Gram-Negative Pathogens

**Figure 1:**
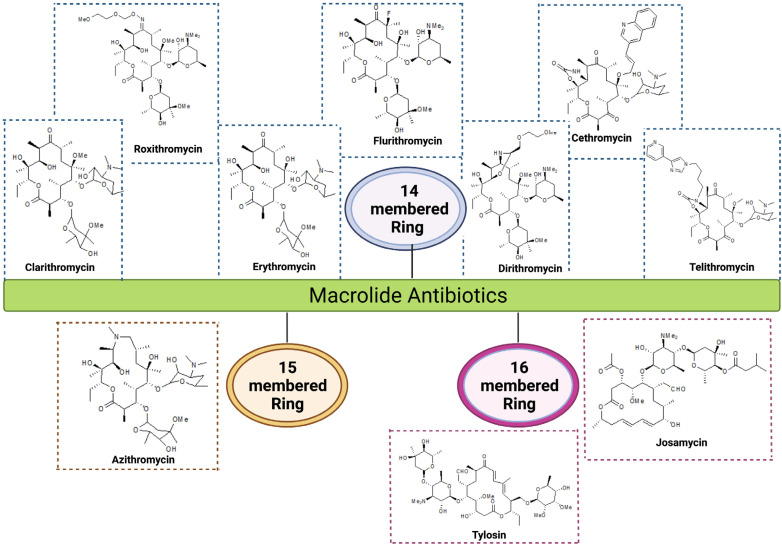
Chemical structures of macrolide antibiotics categorized based on the significant macrocyclic lactone ring structure. The 14-membered (Erythromycin, Clarithromycin, Roxithromycin, Flurithromycin, Dirithromycin, Cethromycin, Telithromycin); 15-membered (Azithromycin); and 16-membered (Tylosin and Josamycin) macrolides.

Macrolides are one of the most frequently used antibiotics, with azithromycin being at the top in many countries around the world [10,11]. Macrolides are classified based on their macro-lactone skeleton, and have either 14-, 15- or 16-membered rings (Figure 1). Macrolides are especially known for their enhanced activity against Gram-positive bacterial infections. However, this group of antibiotics has also been used to treat multiple Gram-negative infections caused by *Bordetella pertussis*, *Neisseria gonorrhoeae*, *Chlamydia* spp., and *Campylobacter* spp. (Table 1). Further, azithromycin has demonstrated a potential role in disaggregating biofilms produced by non-fermenter Gram-negative pathogen *Pseudomonas* spp. [12,13]. This has opened the door for researchers and clinicians to research the synergistic effect of azithromycin with antipseudomonal antibiotics. Also, azithromycin has shown significant efficacy in the treatment of enterobacterial infections and has been widely used in clinical settings to treat different diarrheagenic and systemic infections caused by several enterobacterial species such as *Shigella* spp. and *Salmonella typhi* [14]. This wide application has caused inappropriate and indiscriminate use of this class of antibiotic, leading to the emergence of resistance against macrolides in different bacterial species worldwide [15]. Bacterial resistance to macrolide antibiotics is mainly observed due to two major reasons, such as the narrow binding affinity of the drug to its target or the efflux out of the macrolides from the bacterial cells. The binding affinity of the drug alters due to the modification of either the bacterial ribosomes or the target modification in the antibiotic, whereas the efflux mechanism appears due to high efflux activity within the cell or an alteration in membrane permeability. Apart from these, drug-inactivating mechanisms are also significant for macrolide resistance and are chiefly observed in Gram-negative pathogens.

### 2.1. Target Modification

The primary reason for macrolide resistance in bacterial pathogens is due to the modifications in the ribosomal target site. The most prominent target site modification is the methylation of 23S rRNA, wherein one or two methyl groups are added to the adenine at the 2058 nucleotide position of 23S rRNA post-transcriptionally. This methylation is mainly carried out by adenine-N6 methyltransferases coded by the erythromycin ribosomal methylase (*erm*) family genes [29]. Apart from the methylation of rRNA, mutations in the ribosomal RNA have also been reported to confer resistance to various macrolides and, amongst them, the mutation at position A2058 or A2059 in the ribosomal RNA is considered a significant one, which leads to alterations in the ribosomal target site and eventually inhibits the binding of macrolide antibiotics [19].

The substitution of 23s rRNA in the A2058 or A2059 positions has been well established for causing macrolide resistance in both Enterobacteriaceae and Gram-positive isolates alike [30]. Apart from these two positions, alterations at A745, A752, U754, G2057, A2032, A2062, A2503, U2609, C2610 or C2611 have also been described in Enterobacteriaceae, which can affect the activity of macrolides [15]. Ribosomal protein alterations such as changes in L4 encoded by the *rplD* gene and L22 encoded by the *rplV* gene have also been identified to play a major role in the development of macrolide resistance in *E. coli* [31]. 

### 2.2. Bacterial Efflux Mechanism towards Macrolide Resistance

The efflux pumps are another significant strategy through which the bacteria evade antibiotic action by minimizing the intracellular concentration of the drug. Several chromosomal and plasmid-encoded efflux proteins have been documented, which can transport specific molecules or a range of substrates from within the cells to outside [32]. Among them, the significant pumps associated with macrolide resistance belong to the major-facilitator-superfamily pump (MFS), ATP-binding cassette (ABC) transporters, and Resistance-Nodulation Division (RND) efflux pump family. The *mef*, an MFS pump which includes MefA and Mef E (macrolide efflux), encoded by the *mef*(A) and *mef*(E) genes, confers resistance to most 14- and 15-membered macrolides [33]. These efflux pump genes are mostly associated with mobile genetic elements and were originally described in the *Streptococcus* species. However, due to the wide dissemination of the MGEs, they are not only found in a variety of Gram-positive genera including *Staphylococcus*, *Corynebacterium* and *Enterococcus* but also in many Gram-negative bacteria such as *Escherichia coli*, *Klebsiella*, *Acinetobacter*, *Bacteroides* and *Neisseria* spp. [34]. Macrolide-streptogramin efflux encoded by *msr*, belonging to the ABC transporters family, was first reported in *S. epidermidis,* and contributes to inducible resistance to erythromycin and type B streptogramins. There are four classes of *msr* proteins, viz. *msr*A, *msr*C, *msr*D, and *msr*E, and all of them confer resistance to 14- and 15-membered macrolide and low-level ketolides. These efflux pumps are distributed in Gram-positive isolates as well as in Gram-negative pathogens such as *Pseudomonas*, *Corynebacterium* spp., and others [22,30]. Recently, a few studies have reported that these msr family proteins also work by demounting the bound macrolide from the ribosome and its activity is not just limited to the efflux of antibiotics [30,35]. Another important macrolide efflux pump is the MacAB-TolC of ABC Transporter family, which is a tripartite efflux pump system consisting of the inner membrane transporter MacB, the membrane fusion protein MacA, and the outer membrane channel protein TolC. This tripartite efflux system plays a significant role in the active extrusion of macrolides in *E. coli* and other Gram-negative pathogens [36,37]. Also, the *mef*/*mel* efflux pump system associated with the mega (macrolide efflux genetic assembly) element has emerged as a major resistance mechanism in *S. pneumoniae* and other Gram-positive pathogens. This mega element carries an operon with both *mefE* and *mel* genes in adjacent positions conferring high-level macrolide resistance [38,39,40].

AcrAB-TolC-like RND-type efflux pumps also play a substantial role in macrolide resistance and are majorly present in the Gram-negative enterobacterial isolates. The effect of AcrAB-TolC in the extrusion of macrolides is due to the development of mutants for the expression of genes encoding this efflux pump [41]. Apart from AcrAB-TolC, other important RND efflux systems responsible for the extrusion of macrolides from bacterial cells include MdtEF-TolC in *E. coli*, MexAB-OprM, MexCD-OprJ and MexXY in *P. aeruginosa*, AdeABC in *A. baumannii*, and CmeABC in *Campylobacter jejuni* [38,42,43,44]. Recently, it has been reported that the overexpression of these pumps due to the mutation within RND-type exporters enhances macrolide resistance by increasing the efflux activity of the pump proteins. These emerging mutants significantly enhance the ability of the efflux pumps, resulting in higher MIC towards erythromycin and azithromycin in many Gram-negative pathogens such as *Salmonella* spp. (AcrB), *N. gonorrhoeae* (MtrD), *E. coli* (AcrB), and *Legionella pneumophila* (LpeB) [41]. Thus, these efflux pumps in bacterial isolates decrease antibiotic efficacy many folds and are a major cause of concern for clinicians.

### 2.3. Enzymatic Macrolide Inactivation

The enzymatic degradation of macrolide antibiotics is another mode of resistance mechanism carried out by two different classes of enzymes, viz. macrolide phosphotransferases and macrolide esterases. Phosphotransferases are macrolide-inactivating enzymes commonly reported in bacterial pathogens. To date, seven macrolide phosphotransferases have been described encoded by *mph*A, *mph*B, *mph*C, *mph*D, *mph*E, *mph*F, and *mph*G. Amongst them, *mph*A is the most prevalent in the Enterobacteriaceae family [45]. This enzyme alters the structure of 14-, 15-, and 16-membered lactone rings of macrolide by introducing phosphate to the 2’ hydroxyl group of the macrolide amino sugar, which eventually inhibits the interaction of the antibiotic with A2058.

The hydrolytic inactivation of macrolide can also be mediated by esterase enzymes. EreA encoded by the *ere*A gene has been also identified to be widespread in enterobacterial isolates and is highly potent in hydrolyzing the majority of known macrolides [46]. After the first discovery of EreA in *E. coli*, subsequent reports have revealed different macrolide esterase enzymes, viz. EreB, C and D, in a wide range of bacterial pathogens like *K. pneumoniae*, *Vibrio cholerae*, and *Pseudomonas* spp. [46]. EreC is a close homologue (90.2% sequence similarity) of EreA which could efficiently hydrolyze the majority of the 14-membered macrolides, and azithromycin among the 15-membered rings [47,48]. Currently, there is limited information on the specific substrates of these erythromycin esterases. However, in silico modeling and molecular docking studies of erythromycin with EreC have revealed the molecular mechanism of action of these esterases in hydrolyzing macrolide antibiotics [46,48]. The presence of histidine at the 50th amino acid position of the esterases was identified to play an important role in the cleavage of the ester bond at the macrolactone ring of macrolides, rendering them inactive [46]. The presence of these drug-inactivating enzymes thus drastically reduces the potency of macrolides, making the bacteria resistant towards the antibiotic.

## 3. Approaches to Tackle Antibiotic Resistance with Special Emphasis on Macrolides 

This section of the review discusses the various therapeutic modalities that have been devised to combat macrolide resistance and recapitulates various antibiotic-associated and non-antibiotic-based strategies. 

### 3.1. Non-Antibiotic Approaches

Some of the non-antibiotic-based strategies to fight bacterial resistance include Fecal Microbiota Transplant (FMT) [49], bacteriophage therapy [50,51], clustered regularly interspaced short palindromic repeats (CRISPR) CAS-mediated genome editing [52], quorum quenching technologies [53], stem-cell-derived antimicrobial peptides [54], immunotherapeutic and vaccines. Clarithromycin-resistant *Helicobacter pylori* has become a global concern considering the unavailability of any effective medication against it. The *H. pylori* infection is aggravated by its resistance to even second-generation macrolides. Presently, two candidate vaccines, viz. *H. pylori* surface antigens and the gastric cancer vaccine, are active against this high-priority clarithromycin-resistant *H. pylori* and are in pre-clinical development. These vaccines are likely to combat macrolide-resistant *H. pylori* infection and may aid in improving patient outcomes and the prevention of hospitalizations (who. int) [55]. Additionally, a number of vaccines in mid-stage clinical research have the potential to stop infections caused by resistant bacterial pathogens, such as pathogenic *E. coli*, *A. baumannii*, *P. aeruginosa*, *K. pneumoniae*, and *Clostridium difficile* (Phase II), and *Salmonella typhi* (Phase II), Group B *Streptococcus* (Phase II) and *Haemophilus influenzae* [56]. The use of pneumococcal conjugate vaccines (PCV) has been found to be a successful intervention in lowering the incidence of disease caused by macrolide-resistant pneumococcal serotypes [57,58].

### 3.2. Antibiotic-Associated Strategies

**Figure 2:**
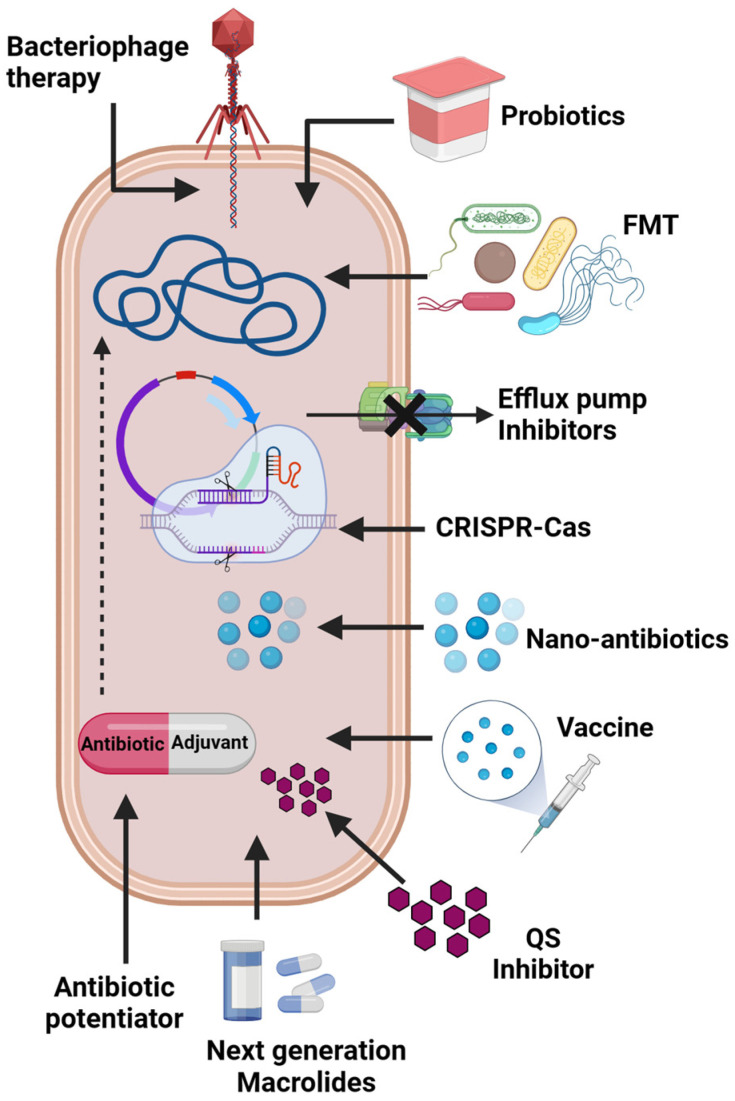
Schematic representation of various antibiotic-associated (antibiotic potentiator, next-generation macrolides) and non-antibiotic (vaccine, Clustered regularly interspaced palindromic repeats-CRISPR-associated protein (CRISPR-CAS), probiotics, phage therapy, Quorum sensing (QS) inhibitors, Fecal microbiota transplantation (FMT) strategies for combating resistant bacterial pathogens.

This includes nano-antibiotics, combination medication therapy, and macrolide potentiation. Nano-antibiotics are encapsulated antibiotic molecules with a size of <100 nm and are one of the growing strategies to counteract the surge of resistant pathogens. In a recent study, the efficiency of clarithromycin nanocrystals towards *H. pylori* has also been investigated and these were found to be effective [59]. The nanocrystals were identified to increase the bioavailability of the drug at the specific site of action compared to other forms of antibiotics, like powder [60]. Another study revealed the enhanced activity of azithromycin nanoparticles (antibiotic-loaded with poly-lactide-co-glycolides) against several clinical pathogens such as *E. coli*, *H. influenza*, and *S. aureus* [61]. Moreover, combination drug therapy using macrolide in conjunction with β-lactams has been reported to treat community-acquired pneumonia (CAP), showing a lower mortality rate and a higher systemic inflammatory response [62]. Also, peptidal antibiotic colistin has been identified to have synergy with macrolide antibiotics and this combination has been identified to be effective against pathogenic Gram-negative rods like *A. baumannii*, *P. aeruginosa* and *K. pneumoniae* [63]. Furthermore, the development of the next-generation macrolide antibiotic solithromycin can be used to combat macrolide resistance. This fourth-generation FDA-approved drug is accepted for intravenous and oral formulations for the treatment of community-acquired bacterial pneumonia and is active against aerobic and anaerobic Gram-positive cocci [64]. Tulathromycin and Gamithromycin are also two next-generation semi-synthetic 15-membered macrolides which are, today, efficiently used against bacterial respiratory infections among veterinary animals [65]. Another important antibiotic-associated strategy through which the efficacy of macrolide antibiotics is enhanced is the combinatorial use of antibiotic potentiators. Antibiotic potentiators are one of the most promising approaches to combat AMR because of the broader coverage, cost-effectiveness and versatility of the approach. The different antibiotic-associated and non-antibiotic-based strategies used for combating macrolide resistance are schematically represented in Figure 2.

#### 3.2.1. Antibiotic Potentiators and Exploration of Their Diverse Mechanisms of Action 

Antibiotic potentiators, also known as adjuvants, are active compounds that have no or little antibacterial activity, but, when combined with antibiotics, can expand the spectrum and enhance the activity of the existing antibiotic against pathogenic bacteria. This renders the resistant bacteria vulnerable to antibiotics again. The high frequency of infections caused by resistant bacteria and the low pace of the discovery of new and effective antimicrobials threaten the future of our healthcare system. So, the best strategy is to increase the efficiency and potentiate the effectiveness of the existing obsolete drugs and make them more useful, which is also economical. Recently, there has been a surge in studies focusing on screening and development of compounds that have antibiotic potentiation ability. Many existing drugs which increase the permeability of the outer membrane and reduce the frequency of the spontaneous resistance for the partner antibiotic have been repurposed to check their potentiation activity [66,67,68]. The major mechanisms through which an antibiotic potentiator acts are as follows: (i) inhibiting antibiotic efflux pump, called efflux pump inhibition (EPI); (ii) inhibiting the drug modifying enzyme; and (iii) increasing the membrane permeability, thereby allowing antibiotic penetration into the bacterial cells. 

##### Efflux Pump Inhibitors

As efflux pumps are considered to be an expeditious and efficient resistant strategy in bacteria, the identification of efflux pump inhibitors (EPI) is a popular approach to inhibit drug expulsion from bacterial cells [69]. The efflux pumps present in the bacterial outer membrane expel the antibiotics, limiting the concentration of antibiotics in the bacterial foci, and preventing its action at the target site. The EPI molecule can be used in combination with antibiotics to enhance their activity against bacterial efflux system and these inhibitors work by blocking the function of the drug efflux transporters, which can be achieved by inhibiting the driving forces of the transporters or strong binding to efflux transporters themselves. The success rate of EPIs in potentiating macrolide antibiotics is limited; however, an earlier study has reported the use of peptide nucleic acid (PNA) antisense agents to reduce the expression of RND efflux pumps in *C. jejuni*, sensitizing the isolate to ciprofloxacin and erythromycin [70]. Similarly, several efflux pump inhibitors, viz. capsaicin, homoisoflavonoid, peptide nucleic acids and phenylalanine-arginine β-naphthylamide, target different pumps like NorA, RND and CmeABC and promote antimicrobial activity against resistant bacteria [71].

##### Modifying Enzyme Inhibitors

Resistant bacteria produce a diverse range of enzymes which can degrade the antibiotics that were supposed to kill the pathogens. These cellular enzymes can change a drug by transferring the chemical moiety or hydrolyzing it, rendering it ineffective. Modifying enzyme inhibitors comprise a wide variety of chemical compounds that specifically target the bacterial enzymes responsible for the hydrolysis of antibiotics and hence increase the effectiveness of the co-administered antibiotic. β-lactamase inhibitors are the most significant and successful clinically used antibiotic adjuvants which prevent the bacterial degradation of β-lactam antibiotics by inhibiting β-lactamase enzymes [72]. They are also referred to as suicide inactivators of β-lactamase due to their irreversible mechanism of action by forming an irreversible acyl-enzyme complex through a covalent bond throughout the catalysis reaction with β-lactam. The most common β-lactamase inhibitors include clavulanic acid (combined with amoxicillin; Co-amoxiclav), sulbactam (combined with ampicillin; ampicillin–sulbactam), tazobactam (combined with piperacillin; piperacillin–tazobactam), avibactam (ceftazidime–avibactam), and relebactam (imipenem–cilastatin–relebactam) [73]. Though there have been no effective inhibitors of macrolide phosphotransferases or macrolide esterases reported so far, the screening of compounds that can inhibit these enzymes could aid in potentiating macrolide antibiotics.

##### Membrane Permeabilizer

The use of membrane permeabilizers to increase antibiotic uptake has proven to be a successful strategy. Different kinds of compounds like peptides, nanoparticles and small molecules can act as outer membrane (OM) permeabilizers [41,74]. OM permeabilizers are a group of adjuvants which interact and disintegrate the OM of Gram-negative bacteria, leading to an increased permeability and concentration of antibiotics within the cell [75]. Erythromycin has limited activity against Gram-negative bacteria due to its hydrophobic nature and high molecular weight, making it incapable of passing through the OM. However, a recent study revealed that the combination of erythromycin with chemically modified guanidinylated derivatives of polymyxins, named guanidinylated polymyxin (GP), reduces the resistance profile of different bacterial pathogens like *Enterococcus* spp., *E. coli* and *A. baumannii* [76]. It has been demonstrated that guanidinylation transforms polymyxin into effective OM permeabilizers, enhancing the permeability of the outer membrane in Gram-negative bacteria. This combinatorial activity of erythromycin/GP is successfully able to restore the susceptibility of resistant pathogens. Due to the powerful activity of nanoparticles, they are emerging as warheads to counter and combat bacterial drug resistance. Some nanoparticles possess antibacterial activity; however, few interact with the antibiotic and enhance the antibacterial activity of the partner drugs by enhancing the membrane permeability of the pathogen. Metal nanoparticles such as silver nanoparticles and gold nanoparticles, as well as several non-antibiotic nanomaterials like graphene oxide nanosheet (GO), graphene oxide-zinc oxide nanocomposite (GN/ZnO) and zinc oxide nanoparticles (ZnO) at a lower concentration, have been identified to have potentiating activity towards different antibiotics including macrolides [74,77,78].

## 4. Macrolide Potentiators and Their Current Status 

**Figure 3:**
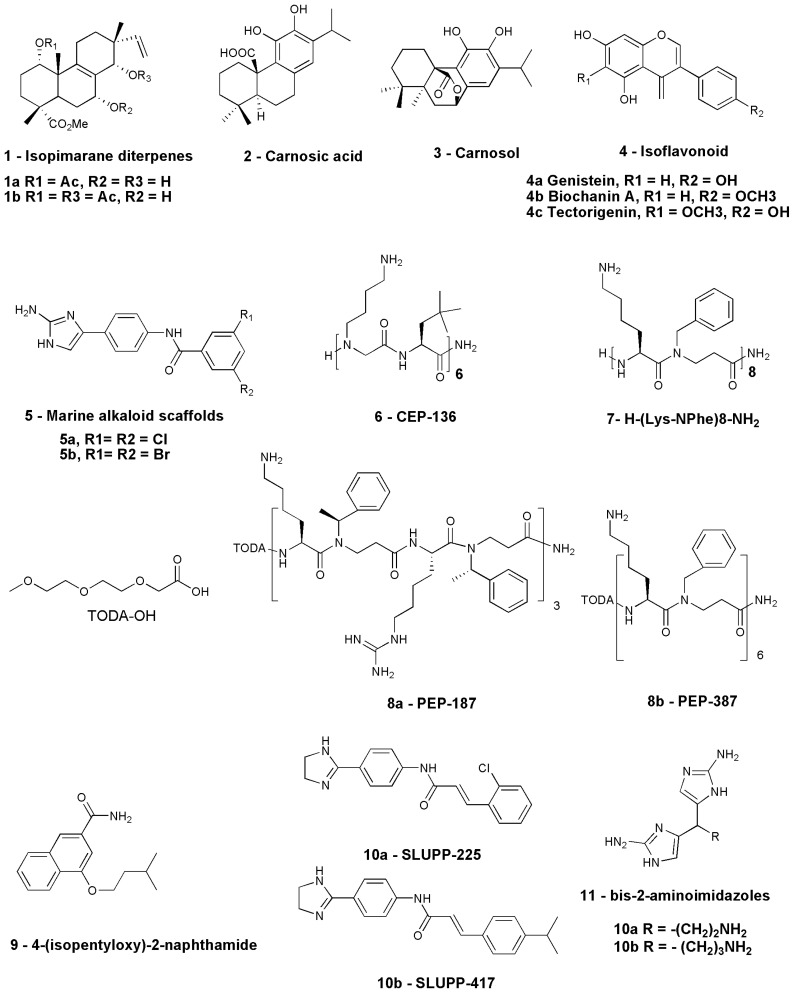
Chemical structures of natural (1–5), peptide-based (6 and 7) and synthetic (8–10) macrolide potentiators.

Antibiotic potentiation is the most promising of the numerous options outlined above to address the growing AMR. Re-purposing compounds with antibiotic potentiation activity is a less expensive and time-efficient alternative compared to developing new antibiotic scaffolds. Certain macrolide antibiotics such as clarithromycin and azithromycin are traditionally not used against Gram-negative pathogens due to their minimal outer-membrane lipopolysaccharide penetration; however, the efficacy and spectrum of such drugs can be improved by using them with a molecule that can facilitate their passage across the bacterial cell by permeabilizing the outer membrane. The quest for macrolide potentiators dates back to the early 1990s. The potentiating effect of normal human serum with macrolide antibiotics was examined in an investigation by Pruul and McDonald in 1992 [79]. The study revealed that the presence of 40% serum decreased the MIC of azithromycin by 26-fold for serum-resistant *E. coli* and 15-fold for *S. aureus* [79]. There have been several reviews in the past highlighting the usefulness of antibiotic adjuvants, particularly for β-lactam antibiotics [80]. However, there have been no or limited reviews on macrolide potentiators. The following sections of this review focus on the natural, synthetic, and peptide-based potentiators studied to date. The chemical structures of a few important macrolide potentiators discussed in this review are presented in Figure 3: 1–10.

### 4.1. Natural Potentiators

Plants have traditionally been a rich source of compounds, and several investigations have been carried out in search of natural compounds with antibiotic potentiation activity [81]. The continuous search for natural products led to the discovery of active extracts of *Lycopus europaeus* that can potentiate macrolide antibiotics. This plant is commonly known as Gipsywort, a perennial plant found on the banks of rivers and canals of the United Kingdom. The plant is also known to have antigonadotropic and antithyrotropic properties due to the presence of phenolic chemicals in it. At 512 µg/mL, two of its isopimarane diterpenes extracts, methyl-1a-acetoxy-7a-14a-dihydroxy-8,15-isopimaradien-18-oate (Figure 3: **1a**) and methyl-1a,14a-diacetoxy-7a-hydroxy-8,15-isopimaradien-18-oate (Figure 3: **1b**), showed potentiation activity of erythromycin by two-fold against macrolide-resistant *S. aureus* isolates expressing msr(A) multidrug efflux pump. At this concentration, these extracts showed no antibacterial activity [82]. Similarly, the chloroform extract of *Rosmarinus officinalis L*., generally known as rosemary, has also been identified to potentiate macrolide antibiotics against macrolide-resistant *S. aureus* strains, decreasing their MIC to 16 µg/mL from 64 µg/mL. Carnosic acid (Figure 3: **2**) and Carnosol (Figure 3: **3**), the two primary components of rosemary, were examined for their potential to boost antibiotic action against resistant strains of *S. aureus*. Carnosic acid (**2**) potentiated the activity of erythromycin eight-fold against msr(A)-expressing *S. aureus* strains and the possible reason for its potentiating ability could be the inhibition of efflux pumps [83]. Natural EPI’s ability to block EmrD-3, a member of the major facilitator superfamily (MFS) transporter family, has been studied in *V. cholerae*. The EmrD-3 efflux inhibitory action of allyl sulfide, a bioactive component of *Allium sativum*, was determined and both *A. sativum* extract and allyl sulfide were discovered to reduce the MICs of several antimicrobials, including erythromycin, by four-fold in bacterial cells expressing EmrD-3 [84]. Further, *Holarrhena antidysenterica*, an ethnobotanical plant mainly used to treat bacterial infections, diarrhea, dysentery, and fever, has also been assessed as a potential EPI. The potentiating ability of Conessine, its principal compound, has been determined with different antibiotics, including erythromycin against wild-type *P. aeruginosa* PAO1 strain K767, MexAB-OprM overexpressed strain K1455, and MexB deleted strain K1523. The overexpression of the MexAB-OprM efflux pump is known to confer resistance to erythromycin and lead to the deletion of *mexB,* resulting in the loss of MexAB-OprM, affecting the susceptibility to antibiotic erythromycin. Conessine significantly reduced the MIC of erythromycin by 4- to 8-fold in contrast to the wild-type strain [85].

In another study including 29 plant species, *Cytisus striatus* showed potential potentiating activity of macrolide antibiotics. An NMR-based metabolomics investigation was carried out to further analyze the chemicals that potentially function as antibiotic potentiators and isoflavonoids present in *Cytisus striatus* were found to enhance the antimicrobial effects of erythromycin against MRSA strains. The 22 isoflavonoids identified were evaluated for their potential as antibiotic adjuvants using the structure–activity relationship (SAR) out of which Genistein (Figure 3: **4a**), Biochanin A (Figure 3: **4b**), and Tectorigenin (Figure 3: **4c**) were identified to potentiate erythromycin activity by reducing MIC by 2- to 8-fold against MRSA strains (Table 2). This research indicates a clear synergy between isoflavonoids and erythromycin, indicating their considerable capacity for being used in the treatment of antibiotic-resistant bacterial infections like MRSA; however, the mechanistic approach is lacking [86].

A study examined the potentiation ability of a wide variety of small molecules from the library based on nitrogen-dense marine alkaloid scaffolds combined with macrolide antibiotics azithromycin, erythromycin, and clarithromycin against *A. baumannii* AB5075 isolate. The research led to the discovery of two compounds of marine alkaloid scaffolds, (Figure 3: **5a** and **5b**) that had previously shown potentiation of β-lactam antibiotics against drug-resistant *A. baumannii* and *P. aeruginosa* (Figure 3). The MIC of most of the macrolide antibiotics in combination with these compounds individually was found to be drastically reduced. The MIC of erythromycin reduced from 32 μg/mL to 4 μg/mL, the effective concentration of azithromycin reduced from 64 μg/mL to 8 μg/mL, and that of clarithromycin from 32 to 0.25 μg/mL. The efficiency of this potentiation strategy for boosting clarithromycin activity has also been investigated in vivo using an *A. baumannii* infection model of *Galleria mellonella* and a high survival rate of the host was observed after a single dosage of a compound **5a** and clarithromycin combination. On analyzing the mode of action, it was identified that the compounds **5a** and **5b** do not disrupt efflux pumps or increase cell membrane permeability via physical disruption; instead, they affect LPS production and its structure by reducing the hydroxylation of C14 and promote lipid A palmitoylation at Cl6 according to GC analysis. Further examination of the LPS composition via gel electrophoresis reveals a structural change in the LPS treated with 5a [87].

**Figure 4:**
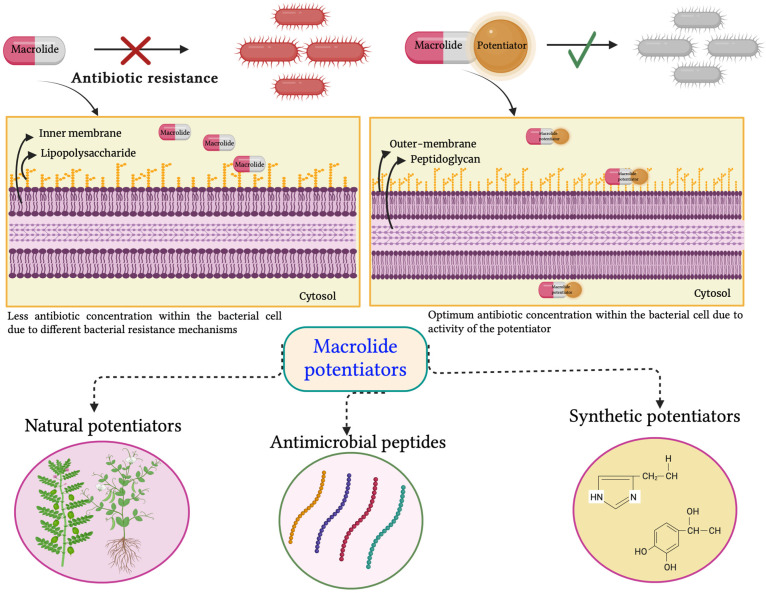
Diagrammatic representation of macrolide potentiation strategy and different types of macrolide potentiators.

A structure–activity relationship (SAR) analysis on the structural alterations produced by compounds **5a** and **5b** to improve the activity of macrolide antibiotics was also conducted to establish the target for these molecules. Further modifications to the core phenyl ring of the compounds resulted in the augmentation of the potentiation activity of clarithromycin by 64- and 32-fold against the *A. baumannii* isolate AB5075. The minimum concentration of the lead compounds for its activity was only 10 and 7.5 μM, respectively, while the required concentration of the original lead compounds was 30 μM. Further modification of the amide linker of the compounds resulted in the development of two new adjuvants consisting of urea that inhibited resistance to clarithromycin at a 7.5 μM concentration in AB5075. The MIC of clarithromycin was reduced 64- and 128-fold [93]. The general mechanism of macrolide potentiators is diagrammatically represented in Figure 4. 

### 4.2. Antimicrobial Peptides as Macrolide Potentiators

Antimicrobial peptides (AMP) are very specific, fast-acting proteinaceous short peptide molecules, with 12–50 amino acid residues, which are known to have antimicrobial, antifungal, antiparasitic, and antiviral properties. These molecules act by disrupting both the outer and inner membranes of the bacterial cell and display reduced cytotoxicity against the mammalian cell membrane. The emergence of resistant microbes, and the growing public concern about antibiotic use, prompted researchers to investigate the ability of AMPs to potentiate antibiotics and thereby re-sensitize resistant pathogens [94]. Peptides and peptidomimetics have displayed their action as potentiators of antibiotics targeting Gram-negative organisms. While most of them act by disrupting the OM, a few of them reduce the efflux pump activity [41]. A handful of antimicrobial peptides have been identified to potentiate macrolide antibiotics and are in different phases of clinical trials, needing further research to understand their efficacy and mechanism of action [41,95]. She et al., recently explored the strong synergistic antimicrobial activity of the SPR741 molecule used in a triple combination with clarithromycin and erythromycin. The triple medication combination demonstrated significant efficacy against extremely drug-resistant *K. pneumoniae* and showed low toxicity in vivo in a neutropenic mouse thigh infection model. The combination also successfully destroyed extremely resistant bacterial biofilms and persisting cells in vitro [96].

Earlier, cathelicidin LL-37, a human AMP, showed improved in vitro antimicrobial efficacy in vitro with daptomycin, a cationic antibiotic. It eradicated β-lactam and vancomycin-resistant *Enterococcus* (VRE) and MRSA in patients [97]. A similar study was performed to validate the efficacy of cathelicidin LL-37 in potentiating azithromycin antibiotics against MDR Gram-negative rods [98]. In combination with cathelicidin LL-37, azithromycin had a strong bactericidal effect against MDR carbapenem-resistant isolates of *K. pneumoniae*, *P. aeruginosa*, and *A. baumannii.* This activity was attributed to the increased penetration of azithromycin when used with peptide LL-37. This implies that azithromycin, which is currently underutilized as the therapy option, can help patients with MDR bacterial infections, mainly when used in conjunction with other molecules. Although earlier studies have shown synergistic antibiotic–peptide interactions, the therapeutic implications of such results have rarely been investigated in depth. 

In yet another study, the screening of thirty-four distinct peptides and four different antibiotics, including erythromycin, against *E. coli* ATCC 25922, four peptides that potentiated erythromycin were discovered. In MDR *K. pneumoniae* ST258 and *E. coli* ST131, the discovered peptides synergized with azithromycin and potentiated clindamycin. The low cytotoxicity of these two peptides (KLWKKWKKWLK-NH2 and GKWKKILGKLIR-NH2) toward eukaryotic cells (IC50 > 50 µM) led to the development and testing of all their D-analogues (D1 and D2) (Table 2). The growth of clinically important *K. pneumoniae*, *E. coli*, and *A. baumannii* strains used in this study was suppressed by lower concentrations of analogues D1 and D2 in conjunction with antibiotic azithromycin. The findings show that combinatorial screening at reduced peptide doses is an effective method for identifying therapeutically important peptide–antibiotic combinations. In vivo, pharmacodynamic/pharmacokinetic, and toxicity investigations are needed to verify the use of the peptides found in these studies with azithromycin antibiotics [88].

Another method is peptidomimetics, which is still in its early phases of development. Peptidomimetics are compounds whose pharmacophores mimic a natural peptide or protein while still interacting with the biological target and delivering the same biological effect. Peptidomimetics were created to solve peptides’ shortcomings, as they are designed to have metabolic stability, high bioavailability, and high receptor affinity and selectivity [99]. Recent research showed that peptidomimetic H-[NLys-tBuAla]6-NH2 (Figure 3: **6**) (CEP-136; NLys = N-(4-aminobutyl) glycine; tBuAla = tert-butylalanine), when tested alone, has low antimicrobial activity against a range of clinically important MDR isolates, including ESBL producers. The peptidomimetic reduced the needed effective bactericidal concentration of different macrolides (azithromycin or clarithromycin) to less than 1 μg/mL in the combination, and this mechanistic insight concludes that the permeabilization of the outer membrane of the Gram-negative bacteria is a strong mechanism to potentiate existing antibiotics including macrolides. Another peptidomimetic, CEP-136 (Figure 3: **6**), displayed low hemolytic activity with no substantial toxicity against mammalian HepG2 cells and did not cause detrimental membrane disruption. In vivo confirmation of CEP-136’s potentiation impact on the treatment of azithromycin in a mouse peritonitis model was possible due to less acute toxicity in healthy mice [40]. In another study, the pairwise screening of 42 synthetic peptidomimetics against the MDR strains of *E. coli* ST131 and *K. pneumoniae* ST258 with the antibiotic azithromycin revealed two α-peptide/β-peptoid hybrids subclasses with fractional inhibitory concentration (FIC) indexes ranging from 0.03 to 0.38. Also, peptidomimetics that augmented erythromycin activity against *E. coli* and clindamycin activity against *K. pneumoniae* were discovered in the same screening with additional antibiotics. Six peptidomimetics were tested against *P. aeruginosa*, and five of these demonstrated antibiotic synergy. H-(Lys-NPhe)8-NH2 (Figure 3: **7**), another promising peptidomimetic molecule, had only a minimal effect on mammalian cell viability and hence displayed the maximum selectivity. At sub-micromolar concentrations of 0.25–0.5 μM, this chemical synergizes with azithromycin, generating sensitivity to the antibiotics at clinically suitable doses in MDR isolates. The identified peptidomimetic molecule and its analogues are attractive prospects for potentiating azithromycin against Gram-negative pathogens [89]. 

In another study, peptidomimetic–macrolide synergy has been analyzed via checkerboard assay in macrolide-resistant clinical strains of *E. coli*. The three peptidomimetics analyzed exhibited synergistic interactions with erythromycin, azithromycin, and tilmicosin against *E. coli*. With exposure to modest concentrations of peptidomimetic (0.5 to 8 mg/mL), the MICs of the three macrolides against these pathogens dropped by 4- to 32-fold. PEP-187 (Figure 3: **8a**) showed faintly larger macrolide potentiation effects than PEP-387 (Figure 3: **8b**) and CEP-136 among the three peptidomimetics examined.

### 4.3. Synthetic Potentiators

The ability of synthetic compounds to potentiate macrolide antibiotics has also been assessed and is discussed in this section. In *E. coli*, the AcrAB-TolC tripartite pump is the primary efflux pump that contributes to resistance. The pump comprises three different proteins. The first one is from the RND superfamily of proteins, the AcrB transporter, which contributes to efflux substrate binding. The second protein is Porin TolC, the channel which bridges the outer membrane and permits substrates to move from the cell into the extracellular space. The third is AcrA, a membrane fusion protein (MFP) that provides a link among them to create a complete channel that precludes the periplasm. AcrB, a substrate-binding protein, has been studied extensively as a potential target for the development of new EPIs [91]. In the quest for AcrB EPI, a molecule, viz. 2-naphthamide, was synthesized. Conventional checkerboard assays revealed that the compound could reverse resistance to antibiotics imparted by the AcrAB-TolC efflux pump. The compound 4-isopentyloxy-2-naphthamide (Figure 3: **9**) was identified to be the most efficient in lowering the erythromycin MIC in sensitive bacterial strains lacking efflux pumps [90]. Another study used in silico virtual screening and experimental screening to find AcrA protein inhibitors. The study resulted in the discovery of two chemicals, SLUPP-225 (Figure 3: **10a**) and SLUPP-417 (Figure 3: **10b**) (Figure 3), which showed promising EPI in *E. coli* cells (Table 2). The compounds identified could traverse the outer membrane, improve efflux suppression, and augment erythromycin activity [89]. *P. aeruginosa* infection is a leading cause of death in cystic fibrosis (CF) patients, and antibiotic therapy remains the mainstay for the treatment of CF caused by *P. aeruginosa*. However, the growing AMR has limited the treatment efficacies and has rendered most antibiotics ineffective. A team of researchers examined a nitrogen-dense heterocycle compound library in search of potentiator compounds that would re-sensitize MDR *P. aeruginosa*. The study resulted in the identification of non-microbicidal bis-2-amino imidazoles (bis-2-AIs) **11a** and **11b** (Figure 3), which could augment the action of azithromycin against *P. aeruginosa* PAO1, a highly inherently resistant strain, and other pseudomonal strains from CF patients [92].

Bis-2-AI **11b** was identified to be the most effective adjuvant in SAR experiments, increasing the activity of azithromycin by a factor of 1024 against PAO1 while presenting no evidence of hemolytic activity far above the effective dosage. Bis-2-AI **11b** made the PAO1 strain sensitive to a variety of antimicrobials, particularly macrolide antibiotic (clarithromycin), doripenem, and rifampin, though it did not potentiate the membrane-active antibiotic colistin. The impact on membrane stability could be the mechanism of action as determined via the BacLight assay; however, further mechanistic experiments for compound **11b** are currently underway to determine whether the observed effect is because of the direct interplay with cell membrane or an indirect impact because of the impaired cell membrane synthesis. Also, iron (Fe) and manganese (Mn) ions were identified to affect the efficacy of the compound, though their exact mode of action was not identified. Additionally, bis-2-AI **11b** was effective in augmenting the activity of azithromycin in the worm model without displaying any toxic or microbicidal activity. The survival rate of worms infected with PAO1 was identified to be 43%, similar to that seen with the positive control penicillin and clavulanic acid.

Apart from this, the recent study by Cui et al. highlighted the potential of phentolamine as a potentiator of macrolide antibiotic. Phentolamine combined with the macrolide antibiotics erythromycin, clarithromycin, and azithromycin indicated a synergistic action against many Gram-negative isolates such as *E. coli*, *S. typhimurium*, *K. pneumoniae*, and *A. baumannii*. The fractional concentration inhibitory indices (FICI) of 0.375 and 0.5 indicated a synergic effect that was consistent with kinetic time-kill assays and a strong synergism has also been observed in the *Galleria mellonella* model [100].

## 5. Hindrances in Taking Macrolide Potentiators from Bench to Bedside and Future Perspectives

The emergence of multi- and pan-drug resistant organisms, colloquially known as “superbugs”, causing illnesses has become an extremely concerning situation worldwide. Furthermore, increased antibiotic use, particularly macrolides like erythromycin or azithromycin, to treat secondary bacterial pneumonia in COVID-19 patients has aggravated macrolide antimicrobial resistance in both Gram-negative and Gram-positive bacteria [101]. Several strategies such as antivirulence strategy, quorum inhibitors, phage cocktails, and CRISPR-Cas technology are being deployed in combination with antibiotics to combat the macrolide resistance threat. However, no such strategy has been identified to be the ultimate option to combat the growing AMR. In recent years, there has been a surge in screening and identification of molecules and compounds that, in combination with obsolete drugs, could revive their lost activity. These studies could identify some very potential antibiotic adjuvants that could potentiate the antibiotic and re-sensitize resistant pathogens. Such studies were also extended to reviving macrolide antibiotics which identified several potential compounds. However, there have been various hindrances in taking macrolide potentiators to clinical use, which are as follows.

### 5.1. Lack of Comprehensive Research and Toxicity Studies

The adjuvant strategy has the potential to overcome macrolide resistance by re-sensitizing resistant Gram-negative and Gram-positive bacteria, including species from the health-threatening ESKAPE category. However, the number of studies on macrolide potentiators and their mechanism of action is very limited. One major reason for this could be the toxicity of macrolide potentiator molecules identified [102,103]. Like the macrolide antibiotic itself, macrolide potentiators, particularly those targeting the efflux pumps, pose significant toxicity concerns, which are a major roadblock for taking such molecules to clinical use. Additionally, because of the lack of definite MIC breakpoints for macrolide antibiotics against different pathogens, many macrolide potentiator compounds are used at high concentrations along with the high dosage of the antibiotic itself to completely inhibit bacterial growth. Another major issue of the macrolide-potentiating molecules identified so far is its acid instability [104]. This makes them suboptimal for oral administration, which further prompts the use of high concentrations of the compound. 

### 5.2. Sensitivity and Specificity

Though there is no high homology between the macrolide resistance enzymes of different bacterial pathogens and human kinases, molecules that act via the resistance enzymes may follow a pathway similar to human serine/threonine (Ser/Thr) or tyrosine (Tyr) kinases and thus show non-specific inhibition of human targets. Thus, the cross-reactivity of macrolide phosphotransferases and macrolide esterase inhibitors with human enzymes represents an obstacle to their use as macrolide adjuvants to rescue macrolide resistance. However, no potential macrolide phosphotransferases and macrolide esterase inhibitors have been identified so far and no in-depth study regarding the structural homology between the bacterial macrolide resistance enzymes and human kinases is available, unlike the aminoglycoside resistance enzymes. Another aspect is the lack of complete understanding of macrolide resistance enzymes, as opposed to β-lactamase enzymes.

### 5.3. Spectrum of Activity and Bacterial Resistance to Macrolide Potentiators 

As the outer membrane of Gram-negative bacteria acts as a barrier to macrolide antibiotics, potentiators that can permeabilize the outer membrane have to be used when macrolides are used against Gram-negative bacteria. However, bacteria on long-term exposure to these macrolide potentiators can acquire resistance towards these molecules which may also be another challenge in antibiotic potentiation strategy. 

Despite the above-mentioned obstacles with potentiators, the scientific community is still working hard to discover more potential macrolide adjuvants. Macrolides are the primary option of medication for *M. avium*, *H. pylori*, Chancroid, and Diphtheria. Treatment of atypical and intracellular pathogenic infections like *Chlamydia* and *Legionella* spp. in parenchymal lower respiratory tract infections or nongonococcal urethritis is among the principal uses for macrolides. The safety of macrolides in β-lactam-allergic individuals, oral bioavailability, acceptability when administered during pregnancy, and efficacy in pediatric settings, as well as in adult and geriatric patients, are additional important benefits of macrolides compared to other drugs. Hence, it is very important to maintain the efficacy of this drug class.

To date, no macrolide potentiator has reached clinical trials, though several natural and synthetic compounds have shown significant macrolide-potentiating activity in vitro. However, the efficacy of very few compounds has been performed in vivo. The efficacies of CEP-136 with azithromycin and nitrogen-dense marine alkaloid compound **5a** with clarithromycin combination were checked in vivo. Comprehensive preclinical studies and optimization via a medicinal chemistry approach are yet to be carried out for these molecules to be further taken for clinical trials. Several peptide molecules have also shown significant macrolide-potentiating activity. Despite this, their clinical usefulness is limited because of undesirable pharmacological characteristics. It is critical to understand the protein homology between bacterial-resistant enzymes and eukaryotic enzymes for a better design of macrolide adjuvants without adverse pharmacological properties. Structure-guided optimization of resistance enzyme inhibitors would be an effective technique to improve the efficacy and reduce off-target effects of compounds and small molecules, paving the way for the identification of macrolide resistance enzyme inhibitors and their human use. Peptidomimetics enables the creation of molecules with finely tuned physicochemical features, resulting in increased antibacterial activity and a better pharmacological profile [105]. More studies into peptide-based potentiators could be beneficial. Multi-omics-assisted identification of resistance determinants and screening of small molecules that bind to these resistant determinants is a straightforward method for macrolide potentiator identification. Also, high-throughput screening techniques such as artificial intelligence and machine learning could be deployed for the fast and less laborious identification of macrolide potentiators.

## 6. Concluding Remarks

The rise in antimicrobial resistance is not only a medical crisis but also a scientific, economic, and interdisciplinary challenge, and it is very crucial to act before it is too late. As AMR is almost becoming an insurmountable challenge, and antibiotics alone are not sufficient to fend off the implacably increasing tide of resistance, it is essential to weaponize antibiotics and use them in medicine against pathogenic strains. The combinatorial activity of an antibiotic and its potentiator provides new insight into the therapeutic option for resistant clinical pathogens. Hence, there is a pressing need to carry out extensive research in the development of effective potentiators of macrolide antibiotics as the resistance against these antibiotics is growing dramatically among Gram-positive and Gram-negative pathogens. In the present review, the authors have recapitulated the different macrolide potentiators, their source of origin, the current clinical status of these molecules, challenges in macrolide potentiator development, and future prospects that can be undertaken for better screening and identification of macrolide potentiators. The search for macrolide inhibitors is a fascinating area of research which is currently expanding. It is not possible to develop a single compound capable of potentiating all classes of macrolides antibiotics due to their significant structural and mechanistic differences. The compounds discussed in the present review are futuristic and capable of potentiating different classes of macrolides antibiotics. The major fallback in the macrolide potentiator discovery research is the lack of in-depth knowledge of resistance mechanisms and systematic and extensive studies on the identified macrolide potentiators. A comprehensive mechanistic understanding of the mode of action of the macrolide potentiators discovered so far can shed light on its opportunities and shortcomings. Further research should be focused on these molecules and experiments to overcome their current limitations. More rigorous in vitro and in vivo studies to understand the safety and efficacy of the newly identified macrolide potentiating compounds have to be performed. Although the development of combinatorial therapy is complicated, considering the proven success of β-lactam potentiators in clinics today, it can be speculated that the use of macrolide potentiators in clinical practice is also a possibility.

## Figures and Tables

**Table 1:** Spectrum of activity of different macrolide antibiotics.

Group	Ring Structure	Molecule	Origin	Target Pathogens	Treatment	Reference
First generation	14-membered	Erythromycin	*Streptomyces erythreus*	Gram-positive bacteria: *Staphylococcus aureus*, *Streptococcus pneumoniae*, and *S. pyogenes* Gram-negative bacteria: *Neisseria meningitis*, *N. gonorrhoeae*, and *Bordetella pertussis*	RTI, skin, soft tissues, urogenital tract and middle ear infections	[16,17]
Second generation	14-membered	Clarithromycin	Semi-synthetic conversion of erythromycin	Gram-positive bacteria: *S. aureus*, *S. pneumoniae*, and *S. pyogenes* Gram-negative bacteria: *Mycoplasma pneumoniae*, *Legionella pneumophila*, and *Chlamydia pneumoniae*, *Helicobacter pylori*, *Pseudomonas aeruginosa*	RTI, chronic inflammation of stomach ulcers, MAC infections in HIV patients	[1,18]
		Roxithromycin	Semi-synthetic derivative of erythromycin	Gram-positive bacteria: *S. aureus*, *S. pyogenes*, *S. pneumoniae*, *Listeria monocytogens* Gram-negative bacteria: *N. meningitidis*, *B. pertussis*, *Haemophilus influenzae*	RTI, skin and soft tissue infection and gastrointestinal infections	[19]
		Flurithromycin	Fluorinated derivative of erythromycin A	*H. pylori*, *Bacteroides forsythus*	Chronic gastritis, periodontal disease	[20,21]
		Dirithromycin	Semi-synthetic derivative of erythromycin	Gram-positive bacteria: *S. aureus*, *S. pneumoniae*, Gram-negative bacteria: *H. influenzae*, *L. pneumophila*, *Moraxella catarrhalis*, *and M. pneumoniae*	Bronchitis, pneumonia, tonsillitis and skin infections	[19]
	15-membered	Azithromycin	Derivative of erythromycin	Gram-positive bacteria: *S. aureus*, *S. pneumoniae* Gram-negative bacteria: *H. influenzae*, *M. catarrhalis*, *C. trachomatis*, *Pseudomonas aeruginosa*, and *H. pylori*	RTI, otitis media, skin and soft tissue infections, gastric and duodenal infections, trachoma eye infections and sexually transmitted diseases	[22,23]
Third generation	14-membered ketolides	Telithromycin	Semi-synthetic derivative of erythromycin	Gram-positive bacteria: *S. pneumoniae* Gram-negative bacteria: *M. pneumoniae*, *C. pneumoniae*, *H. influenzae* and *L. pneumophilia*	Community-acquired respiratory tract infections	[16,24]
		Cethromycin	Derivative of erythromycin	Gram-positive bacteria: macrolide-resistant *S. pneumoniae*, *S. pyogenes* Gram-negative bacteria: *H. influenzae*	Community-acquired pneumonia	[25,26]
	16-membered	Josamycin	*S. narbonensis* var. *josamyceticus*	Gram-positive bacteria: *S. aureus*, *S. pneumoniae*, and *S. pyogenes* Gram-negative bacteria: *H. influenzae*, *M. catarrhalis*, *M. genitalium*, *N. gonorrhea*, *N. meningitidis*	RTI, urethritis	[27]
		Tylosin	*S. fradiae*, *H. influenzae*	*H. influenzae*, Gram-positive pathogens and mycoplasma	Respiratory diseases, mastitis, and dysentery in cattle and other farm animals	[28]

Abbreviations: MAC: Mycobacterium Avium Complex, HIV: Human Immunodeficiency Virus, RTI: Respiratory Tract Infection.

**Table 2:** Natural, synthetic and peptide-based compounds potentiating macrolide antibiotics.

Sl. No.	Compound	Source	Antibiotic in Combination	Organism Tested	Current Status	References
1	**1a**: Methyl-1a-acetoxy-7a-14a-dihydroxy-8,15-isopimaradien-18-oate 1b: Methyl-1a,14a-diacetoxy-7a-hydroxy-8,15-isopimaradien-18-oate	Natural— *L. europaeus*	Erythromycin	*S. aureus* isolates expressing msr(A) multidrug efflux pump	Lab study— in vitro	[82]
2	**2**: Carnosic acid **3**: Carnosol	Natural—*Rosmarinus officinalis* L.	Erythromycin	msr(A)- and NorA-expressing *S. aureus* strain	Lab study— in vitro	[83]
3	Allyl sulfide	Natural—*Allium sativum*	Erythromycin	EmrD-3-expressing *V. cholerae*	Lab study— in vitro	[84]
4	Conessine	Natural—*Holarrhena antidysenterica*	Erythromycin	*P. aeruginosa* PAO1 strain K767, MexAB-OprM overexpressed strain K1455, and MexB deleted strain K1523	Lab study— in vitro	[85]
5	**4a**: Genistein, **4b**: Biochanin A, **4c**: Tectorigenin	*Cytisus striatus*	Erythromycin	MRSA strains	Lab study— in vitro	[86]
6	Compound **5a** and **5b**	Nitrogen-dense marine alkaloid scaffolds	Azithromycin, Erythromycin, Clarithromycin	*A. baumannii* AB5075	Lab study—in vivo using a AB5075 infection model of *Galleria mellonella*	[87]
7	**6**: CEP-136 H-[NLys-tBuAla] 6-NH2	Peptide-based	Azithromycin, Clarithromycin	MDR strains including ESBL-producing isolates	Lab study—in vivo in mouse peritonitis model	[63]
8	KLWKKWKKWLK-NH2 and GKWKKILGKLIR-NH2	Peptide-based	Azithromycin, Erythromycin, Clarithromycin	*K. pneumoniae*, *E. coli*, and *A. baumannii* strains	Lab study— in vitro	[88]
9	**7**: H-(Lys-NPhe)8-NH2	Peptide-based	Azithromycin, Erythromycin, Clindamycin	MDR strain of *E. coli* ST131 and *K. pneumoniae* ST258	Lab study— in vitro	[89]
10	**8**: 4-isopentyloxy-2-naphthamide	Synthetic-2-naphthamide core	Erythromycin	AcrAB-TolC-efflux-pump-expressing strains	Lab study— in vitro	[90]
11	**9a**: SLUPP-225 **9b**: SLUPP-417	Synthetic	Erythromycin	*E. coli*	Lab study— in vitro	[91]
12	**10**: Bis-2-aminoimidazoles (bis-2-AIs)	Synthetic nitrogen-dense heterocycles	Azithromycin, Clarithromycin	*P. aeruginosa*	Lab study— in vivo	[92]

Abbreviations: MRSA: Methicillin-resistant *Staphylococcus aureus*, ST: Sequence type, MDR: Multi-drug resistant, ESBL: Extended-spectrum β-lactamase.

## Data Availability

No new data were created or analyzed in this study. Data sharing is not applicable to this article.
